# Evidence of Late-Summer Mating Readiness and Early Sexual Maturation in Migratory Tree-Roosting Bats Found Dead at Wind Turbines

**DOI:** 10.1371/journal.pone.0047586

**Published:** 2012-10-19

**Authors:** Paul M. Cryan, Joel W. Jameson, Erin F. Baerwald, Craig K. R. Willis, Robert M. R. Barclay, E. Apple Snider, Elizabeth G. Crichton

**Affiliations:** 1 United States Geological Survey, Fort Collins Science Center, Fort Collins, Colorado, United States of America; 2 Department of Biology and Centre for Forest Interdisciplinary Research, University of Winnipeg, Winnipeg, Manitoba, Canada; 3 Department of Biological Sciences, University of Calgary, Calgary, Alberta, Canada; 4 United States Forest Service, Steamboat Springs, Colorado, United States of America; 5 University of Queensland, Gatton, Queensland, Australia; CNRS, Université de Bourgogne, France

## Abstract

Understanding animal mating systems is an important component of their conservation, yet the precise mating times for many species of bats are unknown. The aim of this study was to better understand the details and timing of reproductive events in species of bats that die most frequently at wind turbines in North America, because such information can help inform conservation strategies. We examined the reproductive anatomy of hoary bats (*Lasiurus cinereus*), eastern red bats (*L. borealis*), and silver-haired bats (*Lasionycteris noctivagans*) found dead beneath industrial-scale wind turbines to learn more about when they mate. We evaluated 103 *L. cinereus*, 18 *L. borealis,* and 47 *Ln. noctivagans* from wind energy facilities in the United States and Canada. Histological analysis revealed that most male *L. cinereus* and *L. borealis,* as well as over half the *Ln. noctivagans* examined had sperm in the caudae epididymides by late August, indicating readiness to mate. Testes regression in male hoary bats coincided with enlargement of seminal vesicles and apparent growth of keratinized spines on the glans penis. Seasonality of these processes also suggests that mating could occur during August in *L. cinereus*. Spermatozoa were found in the uterus of an adult female hoary bat collected in September, but not in any other females. Ovaries of all females sampled had growing secondary or tertiary follicles, indicating sexual maturity even in first-year females. *Lasiurus cinereus, L. borealis*, and *Ln. noctivagans* are the only North American temperate bats in which most first-year young of both sexes are known to sexually mature in their first autumn. Our findings provide the first detailed information published on the seasonal timing of mating readiness in these species most affected by wind turbines.

## Introduction

Animals inhabiting seasonally variable environments usually time their sexual activities to coincide with or precede periods when climatic conditions and food resources are favorable for successful reproduction. Despite the critical importance of understanding mating system function for establishing conservation strategies, the details of mating in many animals are unknown [1]. Bats present a particular challenge because their mating periods often extend into seasons when food resources become scarce or unavailable. Approximately 1,100 species of bats occur throughout the world and many are of conservation concern [2], yet the mating systems of relatively few have been well characterized [3].

Mating systems of temperate zone insectivorous bats that have been characterized typically involve reproductive delays with both males and females capable of storing sperm for several months, especially among the Vespertilionidae [4]–[6]. Temperate-zone vespertilionids are unique among mammals because of the pronounced temporal asynchrony of primary (i.e., sperm and ovarian follicle production) and secondary (i.e., heightened libido and copulation) sexual functions. Sperm production by males begins during spring and continues through summer, while females are birthing and raising young [7], [8]. Spermatozoa move from the testes into the epididymides at the end of summer, after which the testes regress and the accessory sex glands enlarge. However, in most species young males do not sexually mature in their first autumn. Copulation usually begins in autumn (September-November) and some species continue mating sporadically through the winter and into spring [9]–[14]. Bats are remarkable among mammals in their ability to store fertile spermatozoa and viable spermatozoa may be stored both within the caudae epididymides of males and the uteri of inseminated females for many months [4]–[6], creating conditions in which successful copulations may occur anytime in the approximately half-year period prior to spring fertilization and gestation in females. This sperm storage allows male bats to mate during times of year when insect prey, necessary to fuel sperm production, is not available. During spring, the accessory sex glands of males begin to shrink, the caudae epididymides empty, and sperm production begins again [15], [16]. At the same time, ovulation, fertilization, and implantation in females mark the start of the spring maternity season [4].

The decoupling of primary from secondary sexual function in vespertilionid bats means that mating rarely coincides with typical externally visible signs of primary sexual function (e.g., maximum testes size) or seasonal increases in resource availability, as is common in other animals. For these reasons, the precise seasonal timing and age at which mating begins in vespertilionid bats can be difficult to determine, because mating might often occur more than half a year before, and sometimes hundreds of kilometers away from, the arrival of pregnant females to resource-rich maternity areas in spring.

Understanding the time of mating and age at first mating in bats is increasingly important in light of mortality of certain species at industrial-scale wind turbines. Migratory vespertilionid bats that rely on trees throughout the year (‘tree bats’) are dying in surprising numbers (tens of thousands per year) at wind turbines [17]–[19]. Carcasses of hoary bats (*Lasiurus cinereus*) are found more often than any other species at turbines in North America and compose an average of about 44% of documented bat fatalities [17]. Closely related eastern red bats (*L. borealis*) average about 28% of fatalities at a continental scale, and sometimes compose more than half of all fatalities at turbine sites in eastern regions of North America [17]. Silver-haired bats (*Lasionycteris noctivagans*) average about 17% all of bat fatalities at a continental scale, and about half of fatalities at certain North American wind facilities [17], [20]. Available evidence indicates that most bats die from collisions with moving blades of turbines, or non-contact depressurization injuries (barotrauma) associated with passing close to moving blades [21]. The vast majority of fatalities occur during late summer and autumn–a period that coincides with both autumn migration and the beginning of presumed mating periods in many of the affected species (e.g., for *Lasiurus* spp. see [22]) [17], [22], [23]. There is not a peak of corresponding magnitude in fatalities of tree bats at turbines during spring migration. This pattern led to the hypothesis that susceptible species of tree bats may rendezvous at visually conspicuous tall structures for mating during autumn migration, and that male bats may die while trying to establish leks (species of *Lasiurus*) and territories (*Ln. noctivagans* and other species) at the tallest ‘trees’ in the landscapes through which they are migrating [22], [24]. So far, the hypothesis that mating is a cause of bat susceptibility to wind turbines is supported only by anecdotal evidence and more data on all aspects of mating by migratory tree bats are critical for evaluating it. An important condition that must apply if mating is a cause of bat susceptibility to turbines is that the majority of tree bats found dead at turbines must be in a state of mating readiness. If this condition is not met, then mating is not an underlying cause of bat fatalities at turbines and is unworthy of the potentially difficult and expensive experiments needed to formally test the hypothesis. Currently there is only sparse information on the timing of reproductive events in species of bats that die most frequently at turbines in North America.

The only prior in-depth study and review of reproduction in tree bats was an unpublished anatomical analysis by Druecker [25]. That work revealed that sperm production in male *L. cinereus* and *Ln. noctivagans* begins in spring, peaks in late summer, and spermatozoa are available in the epididymides of some male *L. cinereus* and *Ln. noctivagans* as early as July and August, respectively. A female *L. cinereus* from New Mexico had been inseminated by early September, and *Ln. noctivagans* was seen copulating in captivity during that month, but spermatozoa were not observed in the one female *Ln. noctivagans* sampled during autumn [25]. The earlier analysis also presented anatomical evidence that a high proportion of first-year male hoary and silver-haired bats achieve sexual maturity before their first winter [25]-a novel finding for North American, temperate-zone vespertilionids. Nonetheless, samples from the months when these species most often die at wind turbines (August-October) were limited in that study (*n = *38 males, 2 females) and were mostly collected in the southwestern United States (US) and Mexico [25]. Reproductive timing in these regions might differ from higher latitudes where the highest fatality rates at wind turbines have been reported in North America [17].

We examined reproductive anatomy of *L. cinereus, L. borealis,* and *Ln. noctivagans* found dead beneath industrial-scale wind turbines to better define the period during which they begin mating and to confirm earlier observations that first-year males can reach sexual maturity during their first autumn. We gathered this information to assess whether the necessary reproductive conditions exist for mating to be possible at turbines during the seasons when fatalities are observed.

## Results

Fifty-seven percent of the female samples we initially collected were unusable because of necrosis, and the ovaries (but not uteri) were too necrotic for analysis in 5 individuals. Twenty-seven percent of the male samples we collected were unusable due to necrosis. These samples were excluded from further analysis. Thus, we evaluated a total of 103 *L. cinereus*, 18 *L. borealis*, and 47 *Ln. noctivagans* (Table 1). Twenty-one percent of *L. cinereus*, 7% of the *L. borealis*, and 64% of *Ln. noctivagans* evaluated were classified as juvenile. Sixty-seven percent of *L. cinereus*, 67% of *L. borealis*, and 51% of *Ln. noctivagans* were male. The majority of samples for both species were found between mid-August and mid-September (Table 1). Different preservation techniques resulted in variable success in preserving samples, but based on our observations we assumed no differences in results among sites due to the opportunistic sampling strategy or bias associated with the success of variable preservation techniques. In general, unfrozen samples fixed in formalin resulted in the best histology specimens.

**Table 1:** Sperm presence in the caudae epididymides (males) and uteri (females) of hoary bats (*Lasiurus cinereus*), eastern red bats (*L. borealis*), and silver-haired bats (*Lasionycteris noctivagans*) found dead beneath wind turbines, as a function of collection period.

Lasiurus cinereus (hoary bat)				
Sex (age)	10 JUL–12 AUG	13 AUG–14 SEP	15 SEP–17 OCT	Total
Male (adult)	70% (n = 19)	90% (42)	100% (9)	89% (70)
Male (juvenile)	0% (2)	60% (10)	–	50% (12)
Female (adult)	0% (2)	0% (8)	100% (1)	9% (11)
Female (juvenile)	0% (1)	0% (8)	0% (1)	0% (10)
**Lasiurus borealis (eastern red bat)**				
**Sex (age)**	**10 JUL–12 AUG**	**13 AUG–14 SEP**	**15 SEP–17 OCT**	**Total**
Male (adult)	100% (n = 10)	100% (4)	100% (1)	100% (15)
Male (juvenile)	100% (1)	–	–	100% (1)
Female (adult)	–	0% (2)	–	0% (2)
Female (juvenile)	–	–	–	–
**Lasionycteris noctivagans (silver-haired bat)**				
**Sex (age)**	**10 JUL–12 AUG**	**13 AUG–14 SEP**	**15 SEP–17 OCT**	**Total**
Male (adult)	–	50% (4)	100% (2)	67% (6)
Male (juvenile)	0% (2)	30% (18)	100% (2)	32% (22)
Female (adult)	0% (2)	0% (4)	0% (3)	0% (9)
Female (juvenile)	0% (1)	0% (9)	–	0% (10)

Listed by sex, age (juvenile  =  born in prior 2–3 months), and showing the percentage with sperm during each period and the total across all periods. Numbers in parentheses indicate number of individuals sampled. Sperm presence in the caudae epididymides of males indicates readiness to mate whereas presence in the uteri of females indicates prior successful copulation.

Spermatozoa were present in the caudae epididymides of 89% adult and 50% of juvenile male *L. cinereus*, 100% of both adult and juvenile male *L. borealis*, and 67% and 32% of adult and juvenile male *Ln. noctivagans* (Table 1). Although samples from certain time periods were limited, prevalence of sperm in adult and juvenile male *L. cinereus* and *Ln. noctivagans* tended to increase from late summer into autumn, whereas all male *L. borealis* collected from mid-July through October had sperm in their epididymides (Table 1).

**Figure 1:**
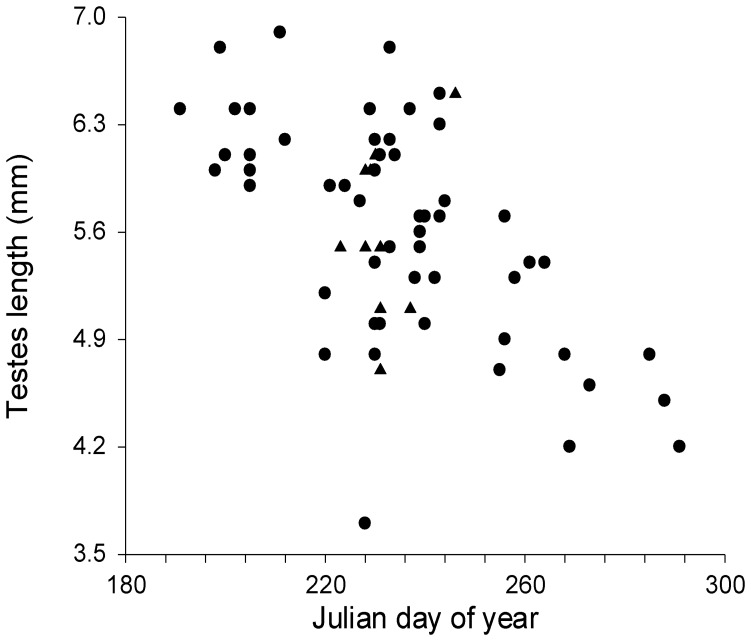
Testes length of a subsample of non-necrotic male hoary bats (*Lasiurus cinereus*) found dead at wind turbines as a function of Julian day of year (from June 29 [Julian day 180] to October 27 [day 300]). Circles represent adults and triangles represent juveniles. The decreasing length of testes with time indicates testicular regression and seasonal termination of primary sexual function (spermatogenesis).

**Figure 2:**
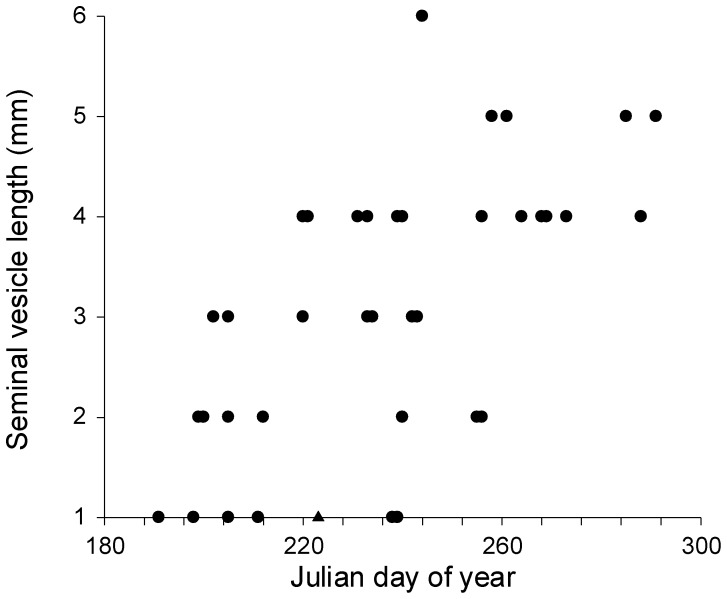
Seminal vesicle length of a subsample of non-necrotic male hoary bats (*Lasiurus cinereus*) found dead at wind turbines as a function of Julian day of year (from June 29 [Julian day 180] to October 27 [day 300]). Circles represent adults and triangles represent juveniles. The increasing length of seminal vesicles with time indicates hypertrophy of accessory glands and an increase in libido and secondary sexual function.

**Figure 3:**
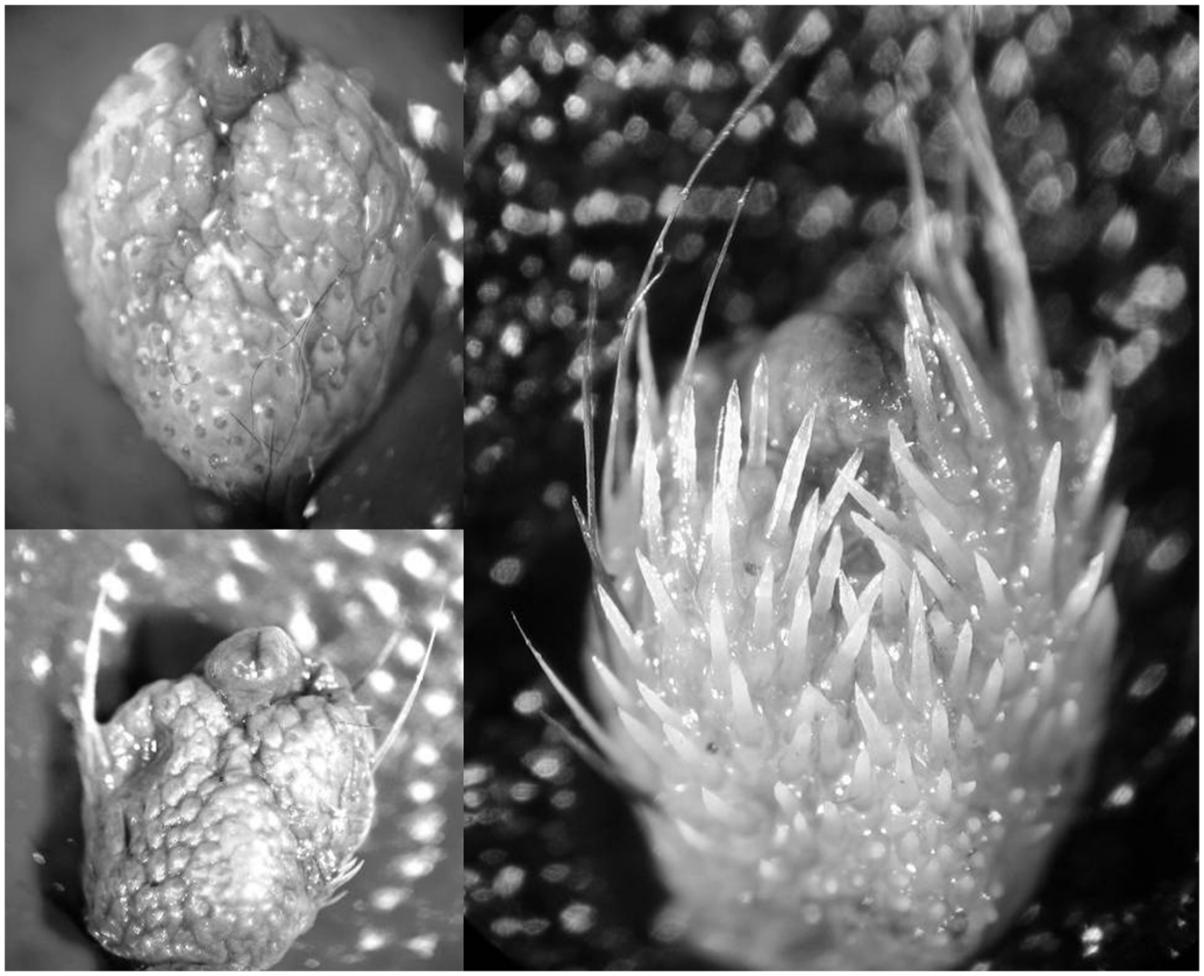
The glans penises of adult male hoary bats (*Lasiurus cinereus*) found dead beneath wind turbines, showing variation in the prevalence and length of well-developed keratinized spines that grow from the surface of the glans and can extend out past its distal tip.

**Figure 4:**
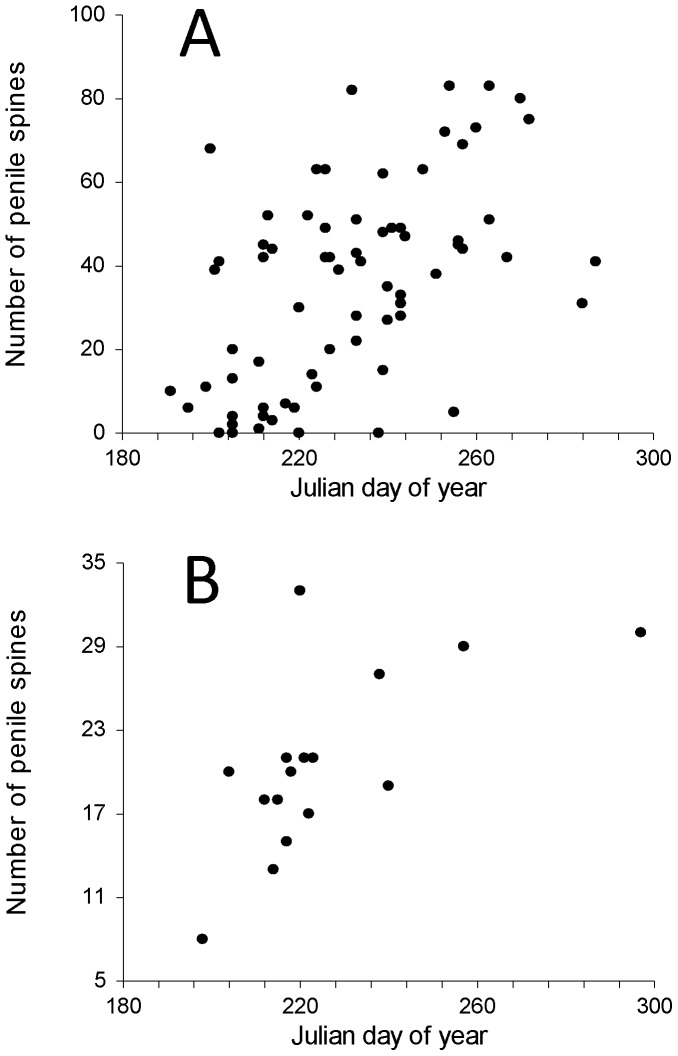
Total number of keratinized spines on the glans penises of (A) hoary bats (*Lasiurus cinereus*) and (B) eastern red bats (*L. borealis*) found dead beneath wind turbines, as a function of Julian day of year (from June 29 [Julian day 180] to October 27 [day 300]).

Length of testes in a subsample of non-necrotic male *L. cinereus* (*n* = 69) ranged from 3.7–6.9 mm and testes length was inversely related to Julian day (Fig. 1; r^2^ = 0.35, p<0.001, t = −5.8), with size decreasing from summer into autumn. Length of testes in a subsample of non-necrotic *Ln. noctivagans* (*n* = 18) ranged from 4.4–6.1 mm and there was no significant relationship between testes length and Julian day, although the sample size was limited (r^2^ = 0.00, p = 0.85, t = −0.2). Length of seminal vesicles in a sample of *L. cinereus* (*n* = 47) ranged from 1–6 mm and there was a significant positive relationship between seminal vesicle length and Julian day (Fig. 2; r^2^ = 0.38, p<0.001, t = 4.9), with length increasing from summer into autumn. Two general types of penile spines were noted in species of *Lasiurus*: longer, slender spines that emerged from the lateral aspects of the glans penis, and shorter, thicker spines that were more evenly distributed across the dorsal and lateral surfaces of the glans (Fig. 3). Significant positive relationships were also observed between Julian day and the number of spines on the glans penis in both *L. cinereus* (r^2^ = 0.31, p<0.001, t = 5.7; Fig. 4A) and *L. borealis* (r^2^ = 0.42, p = 0.007, t = 3.2; Fig. 4B). There were weak correlations between maximum length of spines and Julian day and the relationship was statistically significant for *L. cinereus* (r^2^ = 0.13, p = 0.001, t = 3.3), but not for *L. borealis* (r^2^ = 0.15, p = 0.13, t = 1.6). Spines generally increased in number and length from summer into autumn, although considerable variation was observed and a few males with no epididymal sperm had well-developed, and sometimes darkened (possibly indicating older) spines during early July. The penile spines of 3 juvenile *L. cinereus* we examined were short and curved, whereas those of all other bats examined were straight.

Spermatozoa were observed in the uterus of a single female *L. cinereus* found on 22 September 2008 beneath a turbine in New York. We also noted what may have been sperm in a uterine gland of a juvenile female *L. cinereus* collected on 22 September at the same site, but tissue was too necrotic for confirmation. In the adult female, some spermatozoa were oriented with their heads aligned parallel to each other and perpendicular to the uterine epithelium with which they were closely associated. The uteri of all adult female *L. cinereus* examined (collected between mid-August and mid-September) were glandular or hypertrophied (an indication of hormonal stimulation) whereas the uterus in only 1 of the 9 juvenile *L. cinereus* collected in mid-August showed any signs of stimulation. The uteri in 6 of 8 adult female *Ln. noctivagans* sampled from mid-August to early September showed signs of slight stimulation, but the uterus of only 2 juvenile females of that species found in mid-August and mid-September showed signs of stimulation. Two adult female *L. cinereus* found in mid to late August contained eosinophilic acellular globular masses in the uterine lumen that were suggestive of male accessory gland secretions, but we were unable to locate any signs of spermatozoa in these masses.

All females of each species sampled had growing ovarian follicles. In general, follicular development was more advanced in *L. cinereus* than *Ln. noctivagans*, yet no Graafian follicles were seen in any of the species (Table 2). Samples from female *L. borealis* were too few to assess trends. The ovaries of *Ln. noctivagans* contained larger amounts of interstitial tissue compared with those of *L. cinereus*.

**Table 2:** Most advanced degree of development of ovarian follicles in individual hoary bats (*Lasiurus cinereus*), eastern red bats (*L. borealis*), and silver-haired bats (*Lasionycteris noctivagans*) found dead beneath wind turbines, by species and age group (juvenile  =  born in prior 2–3 months).

	Bilaminar		Multilaminar		Antral	
Species	Adult	Juvenile	Adult	Juvenile	Adult	Juvenile
*Lasiurus cinereus*	1	1	5	1	2	7
*Lasiurus borealis*	–	–	2	–	–	–
*Lasionycteris noctivagans*	1	2	6	8	1	–

See text for description of development stages of follicles.

## Discussion

All of the male *L. borealis* and most of the adult and half of the juvenile male *L. cinereus* we sampled from beneath turbines had moderate to large amounts of spermatozoa stored in their caudae epididymides, indicating that they were capable of fertilizing females. The prevalence of stored spermatozoa in the epididymides of adult and juvenile male *Ln. noctivagans* was lower, but samples from this species were more limited in time, number, and geographic extent. For example, all samples of *Ln. noctivagans* we examined were from sites in New York, or Manitoba and Alberta, Canada, and this species is known to migrate later than *L. cinereus* in the latter region [26]. Our findings confirm the general patterns of male reproductive phenology observed in *L. cinereus* and *Ln. noctivagans* captured in southwestern North America [25]; sperm production generally begins earlier in *L. cinereus* than *Ln. noctivagans* and juvenile males of both species can reach sexual maturity in their first autumn.

In the sample of bats examined during the late 1960’s in southwestern North America, spermatozoa were detected in the caudae epididymides of *L. cinereus* as early as mid-July, but did not appear in the caudae of *Ln. noctivagans* until late August [25]. We also first observed stored epididymal spermatozoa by mid-July in *L. cinereus* and by late August in *Ln. noctivagans*. At a continental scale, fatalities of *L. cinereus* at wind turbines start to increase in number by mid-July, peak during September, then decrease again by October [22]. At many turbine sites, fatalities of hoary bats tend to be biased toward adult males [17]. A speculative explanation for this male bias is that territorial and lekking behaviors of male tree bats seeking mating opportunities at the tallest trees put them at greater risk of fatality at turbines [22], [24]. However, male bias disappears as autumn progresses at the Alberta site we sampled [26]. Seasonal and geographic trends in species differences and sex ratios of bat fatalities at a continental scale have not been assessed, but such information could be valuable toward understanding possible causes of fatality and of male bias. The continental trend in seasonal presence of stored spermatozoa in male *L. cinereus* sampled in this study generally matches the seasonal peak in fatalities of *Lasiurus* spp. at turbines [22].

The lower prevalence of stored sperm in the *Ln. noctivagans* we examined indicates that some male bats die at turbines before they are capable of successful copulation, but this result might be partially biased by the limited number of carcasses we examined; samples of *Ln. noctivagans* were only available from the sites in New York and Canada, were skewed toward juvenile bats, and represented a narrower temporal sampling frame. If male *Ln. noctivagans* die most frequently at turbines prior to mid-August when spermatozoa are beginning to move into the caudae epididymides, it would indicate that mating is not a cause of turbine susceptibility in this species. However, most documented fatalities of *Ln. noctivagans* at the site in Alberta occur after mid-August [26].

The presence of sperm in the caudae epididymides of juvenile *L. cinereus* and *Ln. noctivagans* confirms the unpublished observation [25] that males of both species can become sexually mature in their first autumn. Our observations also prove that juvenile male *L. borealis* become sexually mature in their first autumn. This is unusual for temperate-zone vespertilionid bats [9], [15], [27]. In Europe, low proportions of juvenile males in 3 species are known to become sexually mature in their first autumn (*Plecotus auritus* - [28], [29]; *Myotis daubentonii* - [30], [31]; and *Nyctalus noctula* - [32]). There is also limited evidence that a small proportion of juvenile male *Corynorhinus townsendii* in North America can become sexually mature within 4 months of birth [33]. However, *L. cinereus*, *L. borealis*, and *Ln. noctivagans* are the only three temperate-zone vespertilionids of North America in which a considerable proportion of males are known to become sexually mature within 3 months following birth; although data are lacking, the same may be true for other temperate-zone species of *Lasiurus*. Rapid sexual maturity may have evolved as a response to relatively high natural mortality in species of migratory tree bats, along with other life history traits associated with higher reproductive output (e.g., twins, triplets, quadruplets) [34], [35]. In tropical South America, juvenile males of a congeneric species to the hoary and eastern red bat, the southern yellow bat (*Lasiurus ega*), are also thought to become sexually mature in the first few months after birth [36].

Our data indicate a decreasing trend in testes size of *L. cinereus* found dead at wind turbines from July through October. No such trend was apparent in the limited number of *L. borealis* and *Ln. noctivagans* available to us. Testes of hoary bats found dead beneath turbines in this study showed evidence of a consistent decrease in size earlier than those sampled in southwestern North America by Druecker [25]. Druecker [25] reported an increase in testes length for adult and juvenile *L. cinereus* until early and late August, respectively, before a decline throughout autumn. We observed no such “peak” in carcasses we examined, suggesting that turbines might provide a biased sample toward individuals for which spermatogenesis is nearing completion.

Regression of the testes in vespertilionid bats coincides with arrival of spermatozoa in the caudae epididymides and growth of accessory sex glands, which remain large and secretory throughout winter and stimulate libido [15], [16], [37]. Androgens continue to circulate during the mating season after the testes recrudesce [16] and Leydig cells of atrophied testes likely continue to secrete sufficient quantities of androgen to maintain libido during winter dormancy [9], [37]. Seminal vesicle length of male *L. cinereus* found dead beneath turbines increased during the period of testes recrudescence, indicating that the prevalence of mating readiness in male *L. cinereus* increased from mid-July to September.

This study is the first to report the presence of keratinized spines on the glans penises of *L. cinereus*. We observed a seasonal increase in the prevalence and length of these spines which coincided with testes regression and growth of accessory glands. Similar keratinized spines occur in several orders of mammals [38]–[40], including bats [15], [41]. In *L. borealis*, penile spines are hypothesized to serve as copulatory locks during aerial mating (C. Ritzi, personal communication). In flying insects (*Drosophila bipectinata*), experimental removal of genital spines reduced the ability of males to successfully copulate with females and compete against rival males [42]. Other hypothesized functions of penile spines are sensory feedback during copulation, stimulation of female hormonal state, and/or removal of occlusions such as vaginal plugs [39], [43], [44]. In general, the size of these spines on *Lasiurus* appears to be proportionally larger (sometimes extending to the entire length of the glans) than those of other mammals. Most observations of copulation in species of *Lasiurus* involve aerial couplings in which bats join in mid-air then fall to the ground and remain *in coitus* (reviewed by Cryan 2008; C. Ritzi, personal communication). This behavior has been documented only once near wind turbines [45], but the seasonal increase in prevalence and number of penile spines on hoary bats collected at turbines coincides with sperm availability and accessory gland growth, and seems to be a further indication of mating readiness. Laboratory experiments on rodents demonstrated that growth of penile spines in some species is under androgenic control [44], [46] and we hypothesize that a similar phenomenon occurs in species of *Lasiurus.* Genital size in bats might be used by females as an indication of male genetic quality [47], yet the mechanism by which short-term changes in male condition could produce worthwhile mechanisms or signals of fitness remain elusive. Penis size in common noctule bats (*N. noctula*) varies with body condition and has been hypothesized as a characteristic influencing reproductive fitness [47]. We suggest that if growth in the penile spines of lasiurine bats is proportional to androgen input, then the number and size of penile spines may be a more responsive indication or mechanism of male fitness than other potentially less-responsive morphological features, such as penis size. More or longer penile spines on males of a species that mates in the air may provide clear fitness benefits during copulation.

We found spermatozoa in the uterus of one adult female *L. cinereus* found dead at a turbine on 22 September in New York, and what may have been sperm in a juvenile female collected at the same facility on the same day. We did not observe spermatozoa in any other females sampled. Druecker (1972) observed sperm in the uterus of a female *L. cinereus* collected during early September in New Mexico. These findings demonstrate that successful copulation occurs in at least some *L. cinereus* prior to late September, even at higher latitudes of North America. We found no sperm in the reproductive tracts of female *L. borealis* or *Ln. noctivagans*. Reproductive tracts of female *Ln. noctivagans* collected in southwestern North America did not contain spermatozoa during August or September, but contained abundant spermatozoa in April, indicating that mating in that species most often occurs later than September and possibly during winter [25].

Although we found little direct evidence of recent copulations, we found strong evidence that female bats found dead at turbines were approaching mating readiness. Ovaries of all female *L. cinereus, L. borealis*, and *Ln. noctivagans* had growing follicles, confirming that a high proportion of individuals were reproductively active, and that juvenile females of these species, like juvenile males, reach sexual maturity in their first autumn. The general reproductive cycle of female temperate-zone vespertilionid bats is to enter proestrus and produce Graafian follicles in the ovaries prior to the onset of hibernation, delay ovulation until the end of hibernation, then fertilize the egg with stored spermatozoa from prior copulations [48]–[50]. Although female bats found dead at wind turbines showed few signs of recent copulation, the ubiquitous presence of secondary and early tertiary follicles in both adults and juveniles of each species indicates they were approaching mating readiness.

In the southwestern United States, Graafian follicles were observed in both adult and juvenile female *Ln. noctivagans* in March and April following hibernation [25], but that study did not provide information on ovarian status prior to hibernation. It is not clear whether the absence of Graafian follicles in the female *L. cinereus* and *L. borealis* we examined was the result of the early timing of our sampling or the possibility that females of these species do not produce Graafian follicles prior to autumn migration and (possibly) hibernation. To our knowledge, the chronology of ovarian follicular development has not been described in any species of temperate-zone *Lasiurus* collected from late autumn through winter (October-February). Therefore, it remains unknown whether these bats show the characteristic follicular adaptations of hibernating vespertilionids (i.e., pre-ovulatory follicles indicative of reproductive receptivity) or another pattern of reproductive adaptation. Based on the consistent presence of corpora lutea in many of the female *L. cinereus* sampled in April and early May with growing embryos, Druecker [25] was unable to exclude delayed development or delayed implantation as possibilities. Tertiary follicles with small antra were observed in 53% of the *L. cinereus* we examined, but without additional information on follicular development it is difficult to assess the relative stage of development and whether or not the follicles would be secreting enough estrogen to entice males to mate. In tropical South America, *L. ega* apparently does not produce Graafian follicles prior to tropical ‘winter’, but instead produces tertiary follicles in time for autumn copulation that then grow in size until ovulation in spring [36]. Thus, it appears as though species of *Lasiurus* may be capable of delaying ovulation and storing sperm for several months in the absence of Graafian follicles.

Although sample sizes for females were low in our study, the results do not indicate that frequent and successful copulation occurred at wind turbines immediately prior to death. This finding may indicate that mating does not occur at turbines, because sperm should be present in the reproductive tracts of females visiting tall structures to mate with males. However, if male and female tree bats indeed investigate wind turbines as potential rendezvous sites for mating, then it is possible that females die in the process of approaching turbines prior to finding a mate and successfully copulating. The lack of mature ovarian follicles in females might also be considered evidence that females may not be ready to mate during the period they die at wind turbines, yet mating behavior may precede ovulation and receptivity in vespertilionid bats, as noted above for *L. ega*. Copulation occurs in the hibernator *Myotis lucifugus* during early autumn when the ovaries of females are not yet growing intermediate or large follicles [12], and copulation of *Perimyotis hesperus* begins in September when secondary follicles are becoming tertiary [51]. Additional information on the mating receptivity of female temperate-zone vespertilionids is needed before conclusions can be drawn based on anatomical observations such as ours.

In summary, our findings provide the first detailed published information on the phenology of reproduction in the three species of bats most affected by wind turbines in North America. This information is important because of the suspicion that mating plays a role in the susceptibility of these species to wind turbines. If most tree bats found dead at wind turbines are not consistently found in a state of mating readiness when they die, then mating is not a plausible cause of bat fatalities at turbines and can be dropped from the list of possible underlying causes to be tested with focused studies [23]. We did not test the mating hypothesis in this study, due to our opportunistic sampling strategy, and we did not gather conclusive evidence that tree bats actively mate at wind turbines. However, we did observe that high proportions of males of all three species found beneath turbines showed signs of mating readiness earlier in life and earlier in the year than what is known in most other North American bats, and that signs of male mating readiness increased in conjunction with seasonal patterns of fatalities at turbines. Adult and juvenile females of all three species were clearly approaching mating readiness, although we did not detect sperm in their reproductive tracts before September. We know too little about reproduction in females of any of these species to know whether they may have been receptive to males, but had not yet copulated. Based on our observations, we conclude that mating as a possible cause of tree bat fatalities at wind turbines is worth further pursuing through targeted studies.

The anatomical approach to assessing whether mating occurs at wind turbines has obvious limitations. For example, it is difficult to detect copulation in males or determine if possible mating behaviors that might increase the susceptibility of bats to turbines (e.g., territory or lek establishment and flight displays; see Cryan 2008) occur prior to sperm availability, follicular maturation, or copulation. Additional challenges to this approach include the amount of labor necessary to find fresh carcasses at turbines (e.g., daily searches of areas sometimes encompassing tens of km^2^), rapid decomposition of carcasses in the sun, and bias caused by differential decomposition of male and female reproductive organs (possibly due to proximity of uteri and ovaries to increased microbial activity in abdominal cavity). We were also unable to control for potential site, year, and age effects due to imperfect opportunistic sampling. Although we assumed no such effects, future studies could be designed to test those assumptions. A more targeted approach comparing proportions of individuals showing mating readiness at turbines to those sampled in potentially less-biased ways (e.g., mist netted) may help assess whether reproductively active bats are more likely to die at turbines; existing samples may already be available as fluid-preserved museum specimens. Additional research into the reproductive physiology of migratory tree bats, particularly during winter, will undoubtedly shed important light on the mating systems of these elusive animals and help better assess whether they die at turbines while trying to reproduce.

## Materials and Methods

Carcasses were sampled at 9 different wind energy facilities between 2007 and 2011 in New York and Texas, US, and Alberta and Manitoba, Canada. These wind facilities were highly variable in the number and types of turbines deployed, and surrounding habitats ranged from open prairies to mosaics of forest and agricultural lands. Locations of turbine facilities are available from the authors. Because of the difficulty in systematically obtaining a large number of fresh bat carcasses from any given wind facility over the general period of peak bat fatality in North America (mid-July through October), sampling for this study was opportunistic rather than targeted, resulting in differences in the sites and year that samples were collected and also in the way samples were prepared and preserved. These sampling constraints limited our ability to rule out possible year, site, and age effects. For example, most carcasses of *L. cinereus* found during July came from sites in New York, most found after mid-September came from Texas, and juvenile males of that species were found only during early August of 2008 at 2 sites in New York (*n* = 3) and during a single 15-day period beginning in mid-August of 2007 at the site in Alberta (*n* = 10). Populations of the species examined are thought to be highly migratory [52]. Therefore, because of small sample sizes, variable collection locations and years, and different preservation techniques, we assumed no geographic structure to the populations studied and pooled all samples among sites, years, and age groups for analysis of anatomical measurements. Differences in anatomical in measurements as a function of date were evaluated by linear regression (NCSS, Kaysville, UT) and statistical significance was set at P<0.05.

Carcasses were found during daily to weekly searches beneath turbines generally between the months of about June and October. Only *L. cinereus* were sampled from the Texas site, whereas samples from New York and Canada also included *L. borealis* and *Ln. noctivagans.* Age was determined by visual assessment of ossification in the epiphyseal plates of wing joints [53]. Cylindrical, translucent, hyaline cartilage is visible in the metacarpal-phalangeal joints of the wings of bats for 2–3 months after their birth, herein referred to as ‘juvenile’ bats.

We excised the testes and caudae epididymides of male bats and preserved them in fixative (see below) followed by 95% ethanol. In all of these species, the testes and epididymides are enclosed in a darkly pigmented membrane, the tunica vaginalis [54], which was removed prior to analysis. External measurement or visual assessment of the testes and epididymides through the tunica vaginalis were unreliable for determining reproductive status of males because adipose tissue and the dark, overlying membrane of the tunica obscured the structures [16]. In a subset of non-necrotic samples of *L. cinereus* from the US, we also measured the seminal vesicles *in situ*. Seminal vesicles were measured from the base of the medial cleft to the tip of the longest lobe using a straight ruler. During necropsies we noticed considerable variation in prevalence and length of the conspicuous keratinized spines on the penises of *L. cinereus* and *L. borealis*. We counted the total number and measured the maximum length of spines on the glans penis. The posterior ends of sampled caudae epididymides were removed and prepared as described below for histologic analysis. We removed the uteri and ovaries from carcasses of females and preserved them in fixative prior to analysis.

Male and female reproductive organs were preserved for histology by three different methods because of the opportunistic nature of our sampling: 1) frozen and then fixed in a non-toxic, methanol-based fixative (PROTOCOL SafeFix II, Thermo Fisher Scientific, Waltham, Massachusetts, USA); 2) dissected fresh and fixed in 10% neutral buffered formalin; or 3) frozen in ethanol then transferred to methanol-based or formalin fixative immediately after defrosting. Tissues remained in fixatives for at least 48 hours before being embedded in paraffin, cross-sectioned at 4 µm, and stained with hematoxylin and eosin. We examined 4–5 consecutive transverse sections from each individual sampled.

Histologic analysis of male and female reproductive organs involved noting the presence of sperm in the caudae epididymides of males and uteri of females, as well as the development of ovarian follicles in females. We categorized the most advanced state of follicular development observed in the ovaries of females. Categories of follicular development were: secondary (bilaminar, multilaminar), tertiary (antral), and pre-ovulatory (Graafian). Bilaminar follicles are early growing follicles with 2 layers of cuboidal follicular cells surrounding the oocyte, multilaminar have ≥3 layers of follicular cells, antral follicles have small antra developing among the granulosa cells near the oocyte, and Graafian follicles (not observed in this study) have large fluid-filled antra and eccentric oocytes. Uterine samples were thoroughly scanned for the presence of sperm.
